# Resolutions of Respect to Dr. Charles S. Inglis

**Published:** 1902-03

**Authors:** 


### Obituary.

### RESOLUTIONS OF RESPECT TO DR. CHARLES S. INGLIS.

It is with much regret and sorrow that your Executive Committee, in making its report to you at this the first of our Fall meetings, should find it necessary to record the death of one of the members of this society, in the person of the late Dr. Charles S. Inglis, of Paterson.

In consequence of this sad bereavement, we would offer the following resolutions:

WHEREAS, It has pleased Almighty God, in His wise providence, to remove from our midst our beloved associate and fellow-member of this The Central Dental Association of Northern New Jersey; be it

*Resolved,* That we, the members of this society, do hereby publicly express our sympathy to the bereaved widow and loving helpmate of the deceased; and be it also

*Resolved,* That we lament our loss, since by the death of Dr. Inglis we have personally lost a very dear friend and associate, and the society one of its most promising and useful members; the State one of her favored sons, a professional man of rare attainments, dignified, discreet, brilliant, and attractive; and therefore be it further

*Resolved,* That the above preamble and resolutions be embodied in the minutes of this society, that a copy be transmitted to the widow, Mrs. Inglis, and also that a copy of the same be printed in the Paterson newspapers.

(Signed) FRANK L. HINDLE,
*Chairman,*
P. G. VOEGTLEN,
J. S. VINSON,
F. EDSALL RILEY,
WM. E. TRUEX,
*Executive Committee.*

